# Clinical characteristics and outcomes of Korean patients with sarcoidosis

**DOI:** 10.1038/s41598-021-03175-1

**Published:** 2021-12-08

**Authors:** Ho Jeong Kim, Hyeong Min Kim, Jin Woo Song

**Affiliations:** 1 Department of Pulmonary and Critical Care Medicine, Asan Medical Center, University of Ulsan College of Medicine, 88 Olympic-Ro 43-gil, Songpa-gu, Seoul, 05505 South Korea; 2 University of Ulsan College of Medicine, Seoul, South Korea

**Keywords:** Disease-free survival, Respiratory tract diseases

## Abstract

Sarcoidosis is a systemic granulomatous disorder of unknown cause involving multiple organs. Its clinical presentation and prognosis vary among races. We identified the clinical characteristics and outcomes of Korean patients with sarcoidosis. Clinical data of 367 Korean patients with biopsy-proven sarcoidosis diagnosed in 2001–2017 were retrospectively analyzed. Treatment responses included improvement, stability, or progression based on changes in pulmonary sarcoidosis on chest images. The mean age was 47.4 years, and 67.3% of patients were women. The median follow-up period was 80 months. The highest prevalence was observed in individuals aged 50–59 years (30–39 years in men, 50–59 years in women), and the number of diagnoses showed an increasing trend. Lung involvement was the most common (93.5%), followed by the skin, eyes, and extrathoracic lymph nodes. Among patients with lung involvement and a follow-up period of ≥ 3 months, 66.8%, 31.0%, and 2.2% showed improvement, stability, and progression, respectively. Eleven patients (2.9%) died, and the 5-year survival rate was 99%. The number of diagnosed cases showed an increasing trend, and the mean age at diagnosis was increased compared with that in previous reports. Organ involvement was similar to that of Westerners, although the prognosis appeared better.

## Introduction

Sarcoidosis is a multi-organ inflammatory disorder characterized by the presence of non-caseating granulomas primarily affecting the lungs and lymphatic system. However, its etiology remains unknown^1^. Sarcoidosis occurs worldwide and affects both men and women of all ages and ethnicities. Its incidence and prognosis vary among geographical regions and ethnicities. The highest incidence rates have been reported in African-American and Northern European Caucasian individuals (7.2–35.5 per 100,000 population), while Asians show a relatively lower incidence (0.13–1.01 per 100,000 population)^2,3^. The mortality associated with sarcoidosis is the highest in women of African-American descent (18.5 per one million population)^4^.

There are few previous reports of sarcoidosis in Asian populations, especially South Koreans^5–9^. In previous nationwide population-based studies of the claims database conducted in 2018 and 2020, the incidence of sarcoidosis was 0.81–0.85 per 100,000 population, with the highest rate among those aged 50–59 years^8,9^. In a nationwide population-based study, the annual incidence showed a significant increasing trend from 0.85 in 2009 to 0.97 per 10^5^ population at risk in 2015^8^. The introduction of new diagnostic tools such as endobronchial ultrasound (EBUS) was thought to have partly contributed to the rise in cases^10^. The peak age at diagnosis was the thirties in a prior South Korean study in 2001; this increased to the fifties in a nationwide population-based study in 2018^6,8^. The age-adjusted all-cause mortality rate of patients with sarcoidosis was 9.26–13.1 per 1000 person-years in South Korea, approximately twice that of the general population^7,9^. However, previous studies did not properly reflect the clinical characteristics, including organ involvement, clinical course, and outcome of sarcoidosis in South Koreans. Thus, this study aimed to identify the clinical characteristics and prognosis of South Korean patients with sarcoidosis.

## Methods

### Study population

Between 2001 and 2017, 404 patients with sarcoidosis diagnosed at Asan Medical Center, Seoul, South Korea, were screened. Among them, we excluded 3 patients who were non-South Koreans and 34 whose condition was not confirmed by biopsy. Thus, 367 patients with sarcoidosis were finally included in this study (Supplementary Fig. S1). All patients fulfilled the World Association of Sarcoidosis and Other Granulomatous Diseases (WASOG) diagnostic criteria for sarcoidosis, including having non-caseating granulomas on a biopsy of the involved organs, having compatible clinical features, and the exclusion of other granulomatous diseases with similar features^11^. This study was approved by the Institutional Review Board of Asan Medical Center (2017-1204), which waived the requirement for informed consent because of its retrospective nature. All methods were performed in accordance with the relevant guidelines and regulations.

### Clinical data

The clinical and survival data of all patients were obtained from medical records and/or National Health Insurance Service of Korea records. Spirometry, diffusing capacity of the lung for carbon monoxide (DL_co_), and total lung capacity (TLC) determined using plethysmography were measured according to the American Thoracic Society (ATS)/European Respiratory Society (ERS) recommendations, and the results are expressed as percentages of the normal predicted values^12,13^. Bronchoalveolar lavage (BAL) was performed according to a standardized protocol^14^.

### Organ involvement and clinical course

Each organ’s involvement was defined based on the updated WASOG sarcoidosis organ assessment instrument and confirmed by multidisciplinary discussions based on clinicoradiologic and/or pathologic findings^15^. Organ involvement was assessed using the “at least probable” criteria proposed in the WASOG organ assessment instrument. Lung involvement included intrathoracic lymph node involvement. The chest radiographs were independently reviewed by two pulmonologists (H.J.K and J.W.S). The chest radiographs at the time of diagnosis were staged according to the Scadding radiographical staging system: stage 0, no lung involvement; stage I, bilateral hilar enlargement alone; stage II, bilateral hilar enlargement with lung infiltration; stage III, lung infiltration alone; and stage IV, lung fibrosis. For patients with lung involvement who received treatment, initial outcome was defined as the radiologic response achieved at the end of the treatment. For those who did not receive treatment, it was defined as the radiologic outcome shown at the end of follow-up. Clinical course was analyzed in patients with lung involvement and a follow-up period of ≥ 3 months, considered as the minimum period for evaluating the treatment response^16,17^, and classified as improvement, stability, and progression in a side-by-side comparison. Progression was qualitatively determined as deterioration of existing lesions on the chest x-ray or the occurrence of new lesions, and improvement as reduction of existing lesions. The rest were classified as stability.

### Statistical analyses

Values are given as means ± standard deviations for continuous variables or as numbers and percentages for categorical variables unless otherwise indicated. Student’s t-test or the Mann–Whitney U test was used for continuous data, and Pearson’s chi-squared test or Fisher’s exact test was used for categorical data. The follow-up duration was calculated from the date of diagnosis to the date of death, follow-up loss, or December 31, 2018, whichever came first. Survival was evaluated using the Kaplan–Meier survival analysis. All statistical analyses were performed using SPSS version 26.0 (IBM Co., Armonk, NY, USA). All reported p-values are two sided, and values of p < 0.05 were considered significant.

## Results

### Baseline characteristics

**Figure 1:**
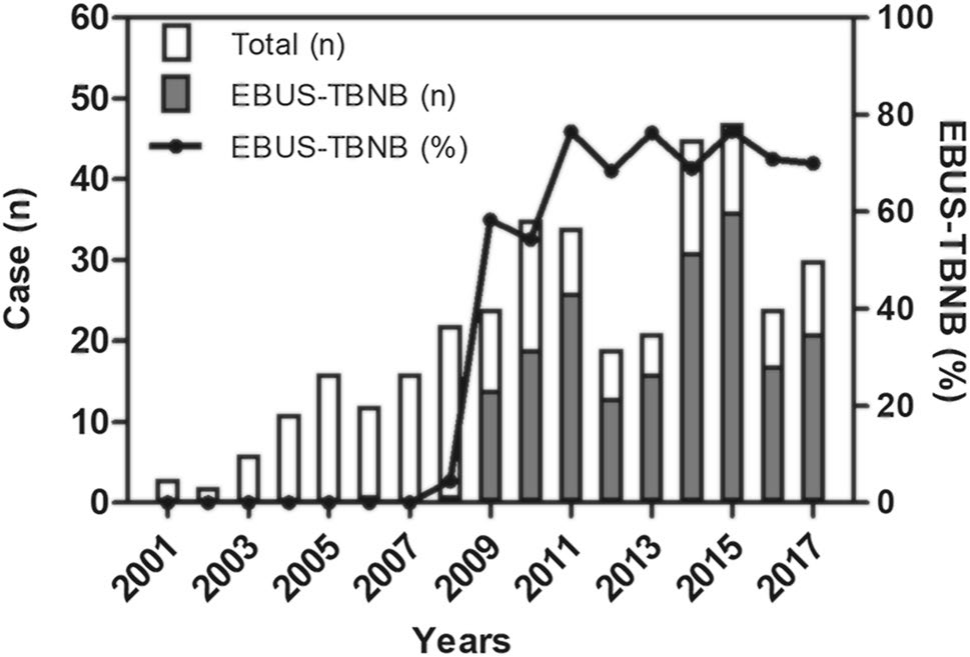
Annual number of patients diagnosed with sarcoidosis in 2001–2017. The white bars represent total patients, and the black bars represent patients diagnosed by EBUS-TBNB. The black lines with dots represent the proportion of patients diagnosed with EBUS-TBNB. EBUS-TBNB, endobronchial ultrasound-guided transbronchial needle biopsy.

**Figure 2:**
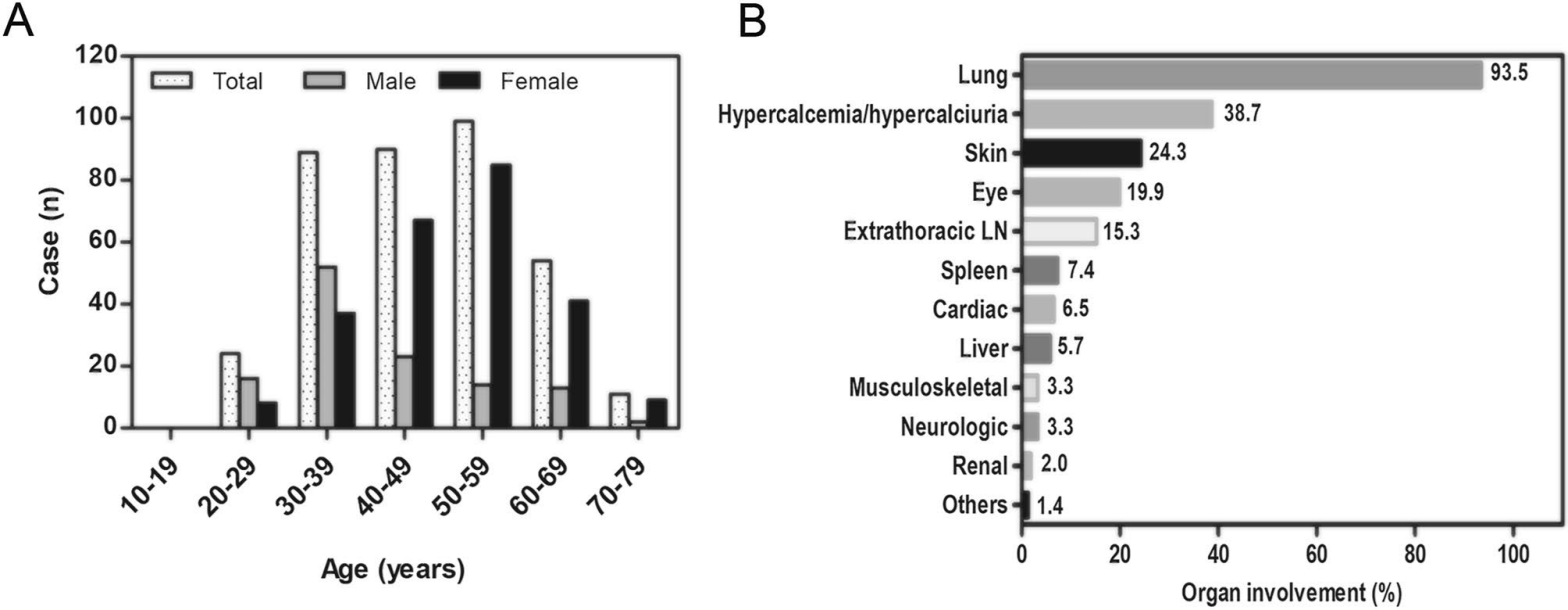
Age distribution and organ involvement of patients with sarcoidosis. (**A**) Age at diagnosis for the total population (white bars), male patients (grey bars), and female patients (black bars). (**B**) Pattern of organ involvement in South Korean patients with sarcoidosis. Each organ’s involvement was defined based on the updated WASOG assessment instrument. WASOG, World Association of Sarcoidosis and Other Granulomatous Diseases.

The mean patient age was 47.4 years, and 67.3% of them were women (female to male ratio: 2.06) (Table 1). The median follow-up period was 80.0 months (interquartile range, 45.0–114.0 months). EBUS transbronchial needle biopsy (EBUS-TBNB) was the most common diagnostic method (52.0%), followed by surgical lung biopsy (25.6%), extrathoracic lymph node biopsy (9.3%), and skin biopsy (9.0%). Supraclavicular lymph node was the most common biopsy site among the extrathoracic lymph nodes. The number of diagnosed sarcoidosis cases showed an increasing trend from 2001 to 2017 (Fig. 1). After 2008, when EBUS-TBNB was introduced at our institution, 301 patients (82.0% of total patients) were diagnosed; among them, 191 (63.5%) were diagnosed by EBUS-TBNB. The most common age at diagnosis was 50–59 years (27.0% of all patients) (Fig. 2A); when stratified by sex, it was 30–39 years among male individuals (44.2%) and 50–59 years among female individuals (34.4%).

**Table 1:** Baseline characteristics of the study population.

Characteristics	
Number of patients	367
Age, years	47.4 ± 12.1
Female sex	247 (67.3)
Ever-smokers	112 (30.5)
**Diagnostic methods**	
EBUS-TBNB	191 (52.0)
Surgical lung biopsy	94 (25.6)
Extrathoracic lymph node biopsy	34 (9.3)
Skin biopsy	33 (9.0)
Transbronchial lung biopsy	9 (2.5)
Musculoskeletal biopsy	4 (1.1)
Heart biopsy	2 (0.5)

EBUS-TBNB, endobronchial ultrasound-guided transbronchial needle biopsy.

### Organ involvement

The lung was the most commonly involved organ (93.5%), followed by the skin (24.3%), eyes (19.9%), and extrathoracic lymph nodes (15.3%). Hypercalcemia or hypercalciuria was present in 38.7% of all patients (Fig. 2B).

Among the patients with lung involvement (n = 342), when classified by the Scadding radiographical staging system, stage II (56.7%) was the most common, followed by stage I (36.8%), stage III (3.8%), and stage IV (2.6%) (Table 2). Among the patients with pulmonary sarcoidosis, 30.6% showed abnormal lung function (obstructive pattern in 7.3%, restrictive pattern in 19.8%, and mixed pattern in 2.3%) with mild impairment. Among patients who underwent BAL (n = 222), 194 (87.4%) showed lymphocytosis (≥ 15% of total nucleated cells in BAL), while 148 (76.7% of patients with lymphocytosis) showed a CD4/CD8 ratio of > 2.5. An atrioventricular or bundle branch block was the most common manifestation (33.3%) in cardiac involvement (n = 24) of sarcoidosis (Table 3). Facial nerve palsy and peripheral neuropathy were the most common manifestation (41.7% each) in neurosarcoidosis (n = 12).

**Table 2:** Pulmonary involvement of patients with sarcoidosis.

Characteristics	
Number of patients	342
**Scadding radiographical staging**	
Stage I	126 (36.8)
Stage II	194 (56.7)
Stage III	13 (3.8)
Stage IV	9 (2.6)
**Pulmonary function test**	
FVC, predicted %	89.8 ± 13.0
FEV_1_, predicted %	87.7 ± 14.3
FEV_1_/FVC	79.0 ± 8.5
DL_co_, predicted %	82.1 ± 14.5
TLC, predicted %	90.3 ± 11.9
**Bronchoalveolar lavage (n = 222)**	
WBCs, /µL	270.9 ± 270.1
Neutrophils, %	2.3 ± 7.2
Lymphocytes, %	39.5 ± 21.1
CD4/CD8 (n = 192)	6.3 ± 5.5
Ever smoker (n = 62)	5.9 ± 5.7
Non-smoker (n = 130)	6.3 ± 5.3

FVC, forced vital cavity; FEV_1,_ forced vital capacity in 1 s; DL_CO_, diffusion capacity of the lung for carbon monoxide; TLC, total lung capacity; WBC, white blood cells.

**Table 3:** Cardiac and neurologic involvement of patients with sarcoidosis.

**Cardiac involvement**	
Number of patients	22
Atrioventricular or bundle branch block	8 (36.4)
Ventricular tachycardia or fibrillation	5 (22.7)
Atrial fibrillation	4 (18.2)
Heart failure	4 (18.2)
Dilated cardiomyopathy	1 (4.5)
**Neurologic involvement**	
Number of patients	12
Facial nerve palsy	5 (41.7)
Peripheral neuropathy	5 (41.7)
Parenchymal brain lesion	1 (8.3)
Hydrocephalus	1 (8.3)

### Clinical course

Of all patients, 58.9% were treated with systemic steroids (Supplementary Table S1). In terms of indications for treatment, pulmonary involvement was the most common (45.4%), followed by neurological involvement (11.6%), hypercalcemia (11.6%), skin (10.2%), eye (9.7%), and cardiac involvement (8.8%), and fatigue (2.8%). The median duration of treatment with systemic steroids was 11.8 months, and the median dose at the start of treatment was 30.0 mg/day (interquartile range, 30.0–40.0 mg/day) based on prednisolone equivalents. However, there was no serious adverse events related to the use of systemic steroids.

**Figure 3:**
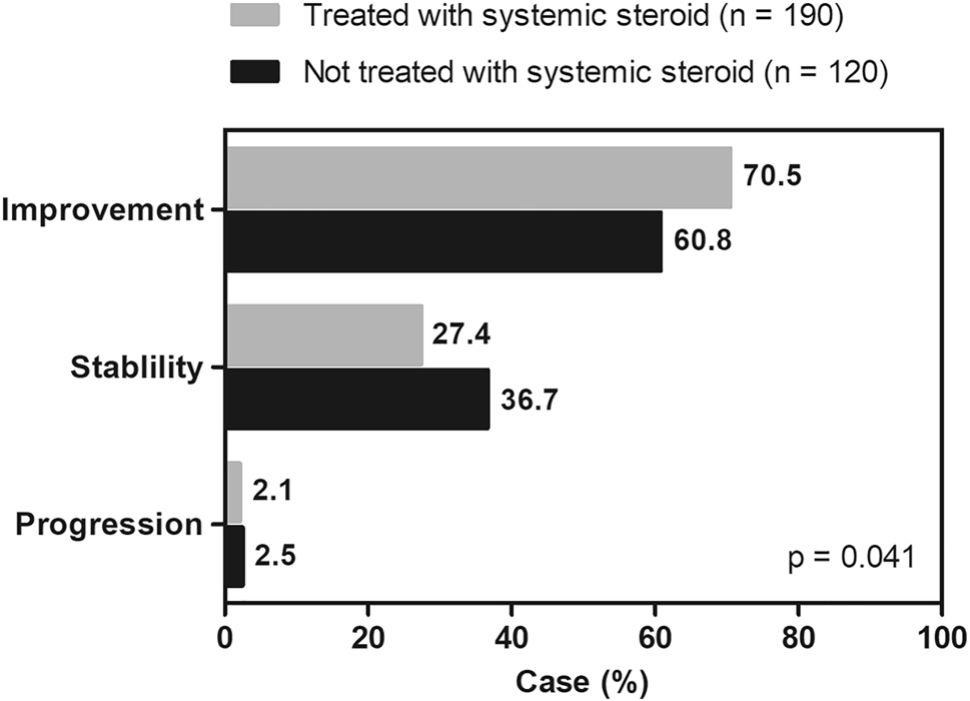
Clinical course of pulmonary sarcoidosis according to treatment status. The grey bars represent patients with pulmonary sarcoidosis treated with systemic steroids, while the black bars represent those without treatment.

Other immunosuppressants were used by 16.1% of the total patients, and 90% were treated with systemic steroids. Methotrexate was the most commonly used immunosuppressant (69.5%), followed by mycophenolate mofetil (20.3%). Clinical course was assessed only in patients with lung involvement and a follow-up period of ≥ 3 months (n = 310); during follow-up, 66.8% showed improvement, 31.0% showed stability, and 2.2% showed progression. When analyzed by treatment status, the patients treated with systemic steroids showed more frequent improvement (70.5% vs. 60.8%, *P* = 0.041) than those without treatment (Fig. 3).

### Survival

**Figure 4:**
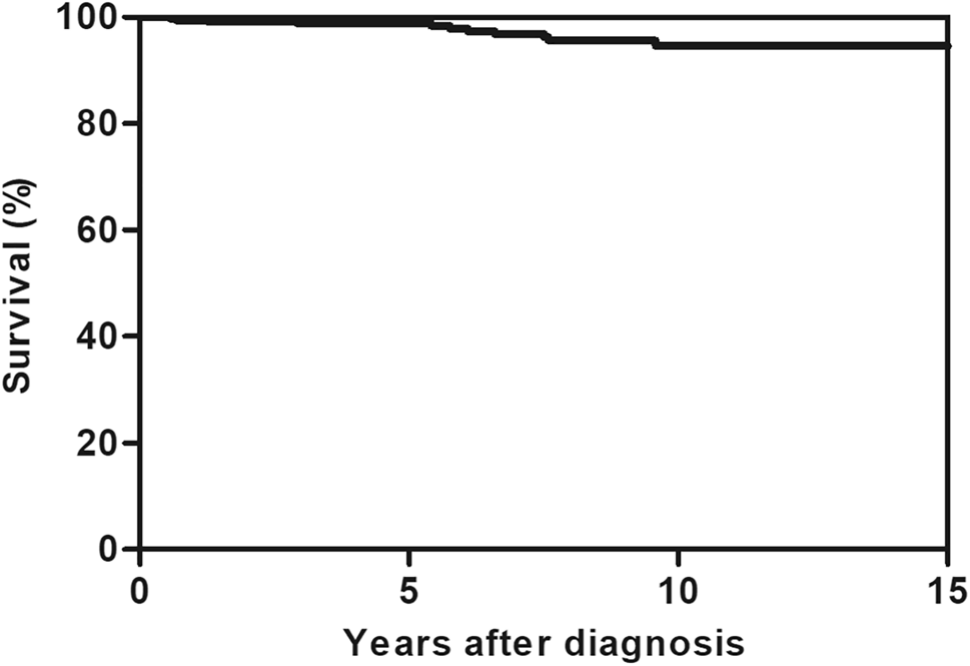
Kaplan–Meier survival estimates of patients with sarcoidosis. The vertical axis represents survival rate, while the horizontal axis represents years after diagnosis.

Eleven patients (3.0% of total patients) died during follow-up, and the most common cause was cancer (n = 4; two patients died of gastrointestinal malignancy and one of lung and endometrial cancer). Three patients died of the progression of sarcoidosis (two with liver involvement and one with neurosarcoidosis). Other causes of death were cerebrovascular disease (n = 2), ischemic heart disease (n = 1), and bacterial infection (n = 1). Among all patients, the 5-year survival rate was 99.0%, while the 10-year survival rate was 95.0% (Fig. 4).

## Discussion

In this study, we retrospectively reviewed South Korean patients diagnosed with sarcoidosis to evaluate their clinical manifestations and prognosis. The number of diagnosed cases of sarcoidosis showed an increasing trend from 2001 to 2017, while the age at the time of diagnosis shifted toward the older age group compared with that reported in the past. Organ involvement was similar to that in Western patients, although treatment response and survival outcome appeared better.

The annual incidence of sarcoidosis reported in a previous South Korean nationwide population-based study showed increasing trends (0.85 per 10^5^ population at risk in 2009 to 0.97 per 10^5^ in 2015)^8^. In our study, the number of patients diagnosed with sarcoidosis also showed an increasing trend from 2001 to 2017. EBUS-TBNB was used as the main tool for diagnosis in 63.0% of the total patients, especially in 81.7% of patients diagnosed after the introduction of EBUS-TBNB in 2008. EBUS-TBNB improved the diagnostic rate of sarcoidosis and might contribute to its increased incidence. Other South Korean studies also suggested that EBUS-TBNB was the dominant tool for diagnosis in 75.8% of all diagnosed cases^10^. Moreover, in South Korea, health screening examinations, including chest computed tomography (CT), are popular, and the number of CT devices in tertiary hospitals has steadily increased since 1995^18^. This might be associated with the increased detection of sarcoidosis in South Korea.

In our study, age distribution at diagnosis shifted toward the older age group compared with that reported in a previous South Korean national survey in 2001 (peak age, 30–39 years)^6^. The increase in age at diagnosis may be due to the aging population. In South Korea, the proportion of senior citizens (those aged > 65 years) was 14.9% of the total population in 2019, an increase of 1.4 times from 11.0% in 2010^19^. Population-based studies in Japan and Sweden also suggested that an aging population is associated with an increase in the age at diagnosis of sarcoidosis^20,21^. When stratified by sex, the age distribution in our study showed different trends; it showed a single peak in the 30–39 and 50–59 years groups in male and female individuals, respectively, similar to the results of Western studies^21–23^. However, our results were not consistent with those of previous South Korean and Japanese studies, which showed biphasic peaks in male individuals in the 30–39 and 60–69 years groups^9,20^. The female dominance observed in our study is comparable to those of other recent South Korean studies (female to male ratio, 2.07 vs. 1.41 to 1.59)^7,8^.

The pattern of organ involvement in our study was similar to that observed in previous South Korean studies conducted by a nationwide survey in 2001 and Western studies; the lung was the most commonly involved organ, followed by the skin and eyes^6,24,25^. Kim et al., in 99 cases of confirmed sarcoidosis in South Korea, reported that stage II was the most common, followed by stages I and III, respectively, and stage IV was found in only 2% of pulmonary sarcoidosis cases, similar to our results^10^. However, in Western patients, stage IV was observed in 5–15% at diagnosis, and 75% of patients with stage IV died of respiratory complications, including pulmonary hypertension (31%) and chronic respiratory failure (25%)^22,26–28^. In a population-based cohort study conducted in the United States (US), the Scadding radiographical stage of pulmonary sarcoidosis at diagnosis was a strong predictor for chronic respiratory impairment defined as forced vital cavity (FVC) or DL_co_ of < 50% and < 40% of the predicted values, respectively, or as the requirement for oxygen supplementation (hazard ratio, 8.36 for stages III and IV versus stage I)^29^. In addition, in a retrospective study using a sarcoidosis cohort in the US, abnormal pulmonary function were observed in 41.1% of the subjects (a restrictive pattern in 21.6%, an obstructive pattern in 17.3%, and a mixed obstructive and restrictive pattern in 2.2%)^30^; however, in our study, 29.4% of the subjects showed abnormal lung function (a restrictive pattern in 19.8%, an obstructive patterns in 7.3%, and a mixed pattern in 2.3%). Moreover, mean lung function was better in our study group (FVC; 89.8% vs. 85.2%, FEV1; 87.7% vs 85.2%)^30^. Based on these results, it can be postulated that sarcoidosis has better prognosis in South Koreans than in Westerners.

During follow-up, in our study, most patients (97.8%) with pulmonary sarcoidosis showed improvement or stability on chest radiography. The ACCESS research group, in a prospective cohort of sarcoidosis diagnosed at 10 centers within the US, reported that 80% of patients with pulmonary sarcoidosis had stable or improved pulmonary function after 2 years of follow-up^31^. Almost 60% of patients with pulmonary sarcoidosis in our study were treated with systemic steroids, and they showed more frequent improvement than those without treatment. In six randomized controlled trials on oral corticosteroid therapy for pulmonary sarcoidosis, treatment with steroids significantly improved symptoms, lung function, and chest radiography results after 3–24 months of treatment, especially in patients with evidence of parenchymal disease on chest radiography^32–37^. A meta-analysis of randomized controlled trials for steroid treatment in patients with sarcoidosis also showed a significant difference in FVC of 4.2% of the predicted values (95% confidence interval [CI] 0.4–7.9) and DL_co_ of 5.7% of predicted values (95% CI 1.0–10.5) compared with untreated patients^38^. However, further studies are required to show whether corticosteroids can prevent lung fibrosis in patients with pulmonary sarcoidosis and improve survival.

In our study, the 5-year survival rate was 99%, higher than that of Western patients. In a study of 1019 patients with sarcoidosis diagnosed between 1991 and 2003 in the United Kingdom, the 5-year survival rate was 93%^23^. In Sweden, a population-based study of 8207 patients with sarcoidosis observed a 95% 5-year survival rate^39^. However, the mortality of patients with sarcoidosis in our study is higher than that of the general population. In a previous South Korean study of claims data, the all-cause mortality rate was 9.26 per 1000 person-years, significantly higher than that of the general population (standardized mortality ratio, 1.91; 95% CI 1.62–2.25)^9^. Pulmonary fibrosis and cardiac involvement were the major causes of death in patients with sarcoidosis^40^. However, in our study, cancer was the most common cause of death, followed by the aggravation of extrathoracic sarcoidosis (neurologic and hepatic involvement). These results are also consistent with the results of the previous South Korean study of Jeon et al.^9^.

Unlike previous South Korean studies that used claims data^5–9^, our research has the advantage of being able to identify patients’ organ involvement patterns, clinical courses, and prognosis. However, it has some limitations. First, it was a retrospective observational study conducted at a single center and included only South Korean patients with sarcoidosis. This may limit the generalizability of our findings. The procedures, such as BAL, were not performed in all patients. Each patient was treated with a different treatment regimen and followed it for a different duration. Therefore, these factors may affect the evaluation of organ involvement and long-term outcomes. However, the baseline characteristics of the patients were similar to those reported in other studies^5–9^. Second, we evaluated the clinical course in the case of pulmonary sarcoidosis alone. However, in most patients, lung involvement was present, and the presence of lung involvement is an important factor in determining patient prognosis. Further research is required of the course of other organ involvement. In addition, many patients did not have serial lung function or symptom data, limiting the analysis of clinical course. Therefore, we used only the radiographic findings to determine the prognosis. Third, for non-pulmonary organ involvement, fewer cases were confirmed by biopsy (20.7%), which is thought to have influenced the lower rate of cardiac or nervous system involvement. However, it was evaluated by applying the WASOG organ assessment instrument. Fourth, the proportion of treated patients was higher than that in other studies, which were usually < 50%^41,42^. Because our institution is a tertiary referral hospital, the condition of the patients included in this study was severe enough to require treatment.

In conclusion, this study suggested that the number of patients diagnosed with sarcoidosis showed increasing trends from 2001 to 2017 and that the diagnosis was most often made in those aged 50–60 years, a shift to the older population, in comparison with that reported previously. Organ involvement in this study population was similar to that in the Western population. Most patients with pulmonary sarcoidosis showed improvement or stability on chest images during follow-up, and the prognosis appeared better than that of Westerners.

## Supplementary Information

Supplementary Information.

## Data Availability

The datasets generated during and/or analyzed during the current study are available from the corresponding author on reasonable request.
