# Supplementary material for: Clinical characteristics and outcomes of Korean patients with sarcoidosis
Source: Sci Rep. 2021 Dec 8;11:23700. doi: 10.1038/s41598-021-03175-1 (PMC8654965; doi:10.1038/s41598-021-03175-1)

**Supplementary information**

**Clinical Characteristics and Outcomes of Korean Patients with Sarcoidosis**

Ho Jeong Kim^1*^, Hyeong Min Kim^2*^, Jin Woo Song^1^

^1^Department of Pulmonary and Critical Care Medicine, Asan Medical Center, University of Ulsan College of Medicine, Seoul, Republic of Korea

^2^University of Ulsan College of Medicine, Seoul, Republic of Korea

* Ho Jeong Kim and Hyeong Min Kim equally contributed to this work as first authors.

**Table S1.** Treatment of patients with sarcoidosis

| Treatment |  |
| --- | --- |
| Systemic steroid, N | 216 |
| Treatment duration, days | 353.5 (162.5 - 819.8) |
| Prednisolone dose, mg/day | 30.0 (30.0 - 40.0) |
| Other immunosuppressants, N | 59 |
| Methotrexate | 41 (69.5) |
| Mycophenolate mofetil | 12 (20.3) |
| Azathioprine | 11 (18.6) |
| Cyclosporin | 2 (3.4) |
| Hydroxychloroquine | 9 (15.3) |

*Data are presented as median (interquartile range) or number (%) of patients, unless otherwise indicated.

N = Number of patients

**Figure legend**

Figure S1. Data source and study population


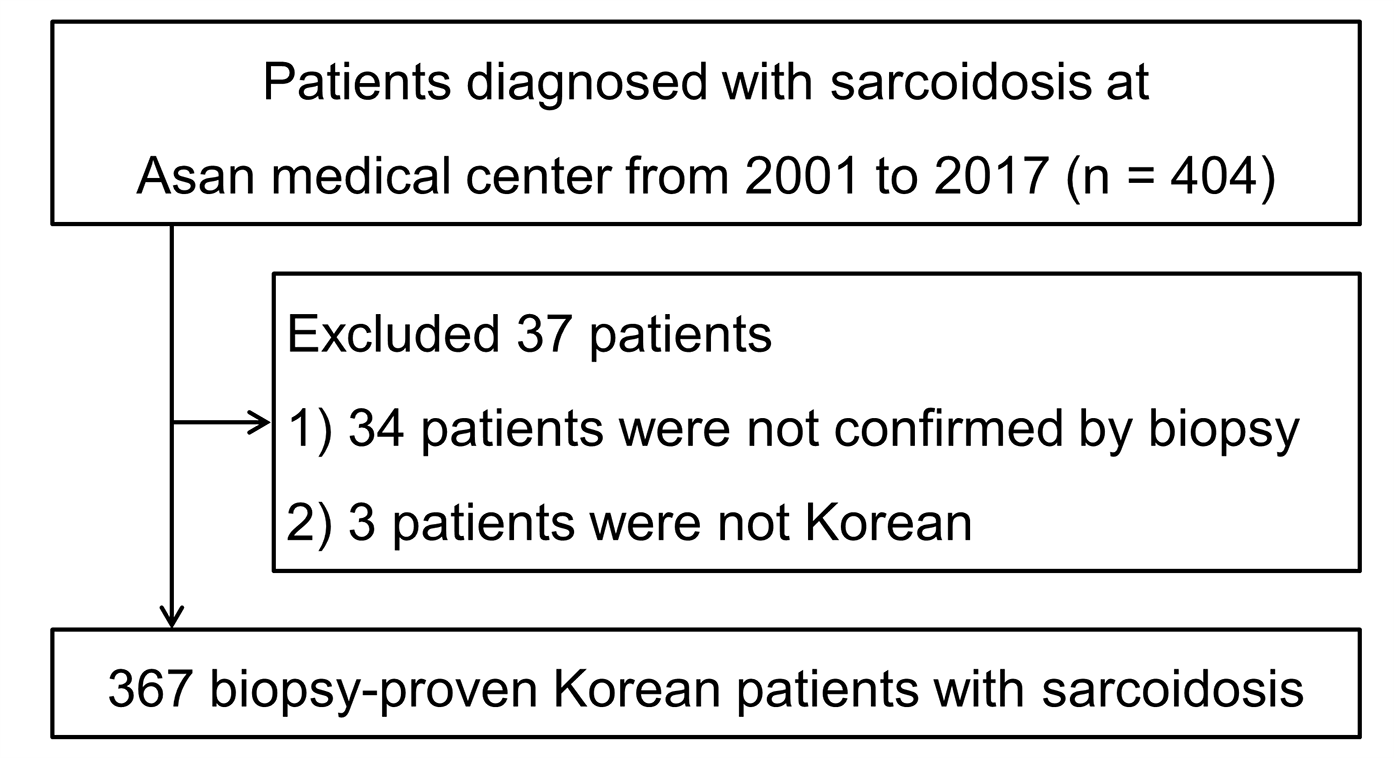

Supplement: Supplementary file 1 — Supplementary Information. [file 41598_2021_3175_MOESM1_ESM.doc]
